# Differences in addiction and recovery gains according to gender – gender barriers and specific differences in overall strengths growth

**DOI:** 10.1186/s13011-022-00444-8

**Published:** 2022-03-14

**Authors:** Valeria Abreu Minero, David Best, Lorna Brown, David Patton, Wouter Vanderplasschen

**Affiliations:** 1 Department of Criminology, College of Business, Law and Social Sciences, University of Derby, Derby, UK; 2 Recovery and Addiction Cluster, Department of Special Needs Education, Ghent University, Ghent, Belgium

**Keywords:** Addiction, Recovery, Gender differences, Strengths, Substance use disorder, Barriers

## Abstract

**Background:**

There is growing evidence on the importance of a gendered understanding of recovery. Gender differences have been reported in relation to the nature and extent of substance use, pathways to and through substance use disorder and recovery capital acquisition and maintenance. There is little existing research on factors associated with recovery capital growth by gender.

**Methods:**

The current paper uses the European Life in Recovery database to assess specific domains of the Strengths and Barriers Recovery Scale (SABRS) that best predict growth of recovery capital amongst people in recovery from drug addiction. The 1313 participants were drawn from the REC-PATH study and recruited by the Recovery Users Network (RUN) from across Europe. Bivariate and multivariate analyses were performed to identify relationships between specific SABRS items and gender, as well as differences in the dimensions of the SABRS scale most likely to predict recovery capital growth by gender.

**Results:**

Between their time in active addiction and in recovery, females show greater growth in strengths, despite females reporting fewer recovery strengths during active addiction than males, and males have greater reductions in barriers to recovery compared to females. Multivariate analyses show that strengths specifically related to prosocial meaningful activities are found to be highly significant for growth of recovery capital amongst males, whereas strengths related to both prosocial meaningful activities and general health management seem particularly relevant for growth of recovery capital amongst females.

**Conclusions:**

We conclude that this further demonstration of gender differences in recovery pathways should suggest gender-specific approaches adopted in recovery community organisations to address these different needs.

## Introduction

Addiction recovery is generally understood as a multi-factorial and non-linear process that takes place over extended periods of time. According to White [31], an ideal definition of recovery would meet six criteria: (a) precision (captures the essential nature and elements of the recovery experience), (b) inclusiveness (encompasses diverse recovery experiences, frameworks, and styles), (c) exclusiveness (filters out phenomena lacking essential recovery ingredients), (d) measurability (facilitates self-assessment, professional evaluation, and scientific study), (e) acceptability (to multiple constituents), and (f) simplicity (elegant in its clarity and conciseness). The Betty Ford Institute Consensus Group [8] definition of recovery differentiates between ‘early recovery’ (of up to one year), ‘sustained recovery’ (of between one and five years) and ‘stable recovery’ (of more than five years), based on diminishing relapse risks with increased recovery time. Both the process of recovery and the time required will vary between individuals [29]. This diversity of experience around recovery has been shown to relate to gender (32). Despite the increasing interest in lived experiences of those in recovery, little is known around the role gender might play in the journey to recovery [19]. There is an increasing need for further exploration around the gendered nature of recovery experiences [11, 24].

Gender plays a crucial role in understanding how individuals progress through the treatment, relapse, and recovery cycle. For example, less than one-third of individuals accessing alcohol and drug treatment in England from 2019 to 2020 were female [25]. Significantly lower numbers of women in treatment populations may reflect differences in barriers women face in accessing treatment, including mental health issues, and caring responsibilities (28 as cited by 1). Yet, Grella and colleagues [16] argued that gender is not only relevant for its impact on the course of substance use initiation, addiction onset, and treatment participation, but also for the outcomes following treatment and recovery.

Increasing evidence suggests that women’s and men’s recovery experiences may be distinct. Research from the US [13], Canada [24], Australia [12] and the UK [2, 3] found that recovery from alcohol and drug problems results in marked improvements across five domains, namely work, finances, legal status, family and social relationships, and employment – but with sufficient local variations to suggest that recovery pathways are not insensitive to local and cultural contexts [3]. More recently, other authors have identified significant gender differences in recovery trajectories. Andersson et al. [1] reported that a greater proportion of females in recovery reported having specific needs in relation to mental health and relationships with children or partners whilst a greater proportion of males disclosed unmet needs around physical health.

Efforts around encapsulating recovery experiences have resulted in the development of a metric for encapsulating recovery progress termed ‘recovery capital’, originally defined as ‘the sum total of one’s resources that can be brought to bear on the initiation and maintenance of substance misuse cessation’ ([4], p. 1972]). A growing interest in the measurement of recovery capital has resulted in the development of the Assessment of Recovery Capital scale (ARC), an instrument designed to measure both strengths and barriers (across ten domains of personal and social recovery capital) [17]. Findings have consistently shown that the recovery journey typically involves the accretion of assets and the reduction of barriers and unmet needs [15], although this does not occur in a linear manner.

Additional studies have used data gathered via the UK Life in Recovery (LiR) survey [5]. A recent innovative study by Best, Vanderplasschen and Nisic [7] quantified the LiR survey, allowing for a more detailed exploration of changes in the recovery journey through the development of the strengths and barriers recovery scale (SABRS). The procedure used by Best et al., [7] entailed dividing relevant LiR items into strengths and deficits questions and generating change measures by subtracting the active addiction scores from recovery measures (e.g., change in involvement in family activities) [7]. All items that had a positive valence (such as “I exercise regularly”) were categorised as Recovery Strengths and all items that had a negative valence (such as “I have been to prison”) were categorised as Recovery Barriers. There were no neutral items. As each item was simply endorsed or not, this allowed a simple tally of recovery strengths and recovery barriers at two time points— “In active addiction” and “In recovery.” A proxy measure of change could then be calculated by subtracting each “In active addiction” composite score from each “In recovery score,” generating overall change scores for Recovery Strengths and Recovery Barriers. Thus, the SABRS instrument enables for not only calculating overall recovery strengths and recovery barriers scores, but also the possibility to determine whether these recovery capital measures differ across stages of the recovery journey [7]. The ARC and SABRS tools were based on the idea that recovery capital is not only something that can be measured, but it can be assessed at various moments as it continues to change over time.

Only a handful of studies have reported on differences relating to the dynamism of recovery capital according to gender. Best et al.’s [7] SABRS scale study showed that whilst males reported significantly more recovery strengths during their time in active addiction, this situation was reversed with females in the recovery period. Further, females not only reported more strengths in recovery compared to males, but also greater growth in strengths in the period from addiction to recovery [7]. This may indicate women’s capacity for developing a diverse range of skills across recovery stages, but this conclusion requires considerably more research and analyses across specific domains and resources. Another study by Best and colleagues [6] showed that the association between living with dependent children and reporting greater gains in recovery capital across the recovery journey is more significant in females compared to males, emphasising important gender differences in relation to the recovery trajectory. Still, there is a scarcity of research identifying which specific features of recovery capital show marked gendered differences across the recovery journey.

The current paper builds on prior work by Best and colleagues [7] on the use of the SABRS scale and findings around notable differences between men and women in their barriers and strengths to recovery and in the process of change over time. Specifically, to better understand the increase of recovery strengths in women compared to men by exploring the domains of recovery capital under which this growth is most likely to occur. Our aim is to add to the granularity of the analysis of gender differences by first exploring significant relationships between gender and both strengths and ongoing barriers to recovery (RQ_1_). Next, we will use findings from the first step in analyses to better understand the specific domains of recovery capital that best predict an increase of recovery strengths separately for women and men (RQ_2_). This study seeks to assess the consistency of gender effects across national contexts by including the RUN sample from a range of European nations. The research questions addressed in this paper are:

RQ_1_ Is there a significant relationship between individual SABRS items and gender at time of recovery?

RQ_2_ Which domains of recovery capital predict greater gains of recovery strengths in women compared to men?

## Methods

### Design and procedure

The paper is based on a convenience sample initially recruited during the REC-PATH study, an EU-funded multi-country and multi-method study on recovery pathways and experiences among persons with a history of illicit drug addiction. Between January and June 2018, the Life in Recovery (LiR) survey was used as a recruitment and screening instrument in the United Kingdom, the Netherlands, and Flanders (Dutch speaking part of Belgium) (*n* = 776). Individuals were also recruited through RUN (Recovered Users Network) in Serbia, Poland, Bosnia and Herzegovina and Spain (*n* = 537) as a supplementary project not included in the main REC-PATH dataset but using the same basic data collection methods following translation into the appropriate languages. The total sample for this study consisted of 1313 participants. More information on the procedure for the REC-PATH [3, 23] and RUN data collection [7] can be found elsewhere.

### Instrument

The development of the strengths and barriers recovery scale (SABRS) aimed to measure positive and negative recovery capital in addiction and in recovery. The original set of 44 items in the LiR survey was reduced to 32 items, consisting of 15 strengths items and 17 deficit items (see Table 1). Each item used a binary (yes/no) response option format, creating a scale of 0–15 for strengths and 0–17 for deficits and thus four score totals per participant (total Recovery Strengths and total Recovery Deficits; each for Active Addiction and in Recovery). These four domain scores allow a “change” analysis to be conducted, where the growth in strengths can be calculated as the total of Recovery Strengths in Recovery minus the total of Recovery Strengths in Active Addiction. We use “change” as a proxy indicator, given we only use a one-time point of data. Indeed, our study uses retrospective recall of past-behaviour using a cross-sectional survey which asks participants about historical and current information to then assess the “change” between the two. Additional information around the development of the SABRS scale and domains that form the LiR has been previously outlined elsewhere [6, 7].

**Table 1:** Final set of included items in the Strengths and Barriers Recovery Scale (SABRS) (*n* = 32)

Recovery Strength Items	Recovery Barrier Items
Exercise regularly	Have untreated emotional or mental health problems
Have a GP	Make regular visits to the emergency room
Have regular dental checks	Regular use of health services
Have good nutrition	Smoke
Take care of your health	Have your driver’s licence revoked
Maintain a driving licence	Drive under the influence of alcohol or drugs
Maintain a bank account	Damage property
Able to pay bills	Been arrested
Maintain stable housing	Been charged with a criminal offence
Remain in steady employment	Been to prison
Further your education or training	Have bad debts
Start your own business	Were unable to pay the bills
Participate in family life	Regularly missed school or work
Plan for the future	Dropped out of school or work
Volunteer	Fired or suspended from work
	Lose custody of children
	Experience family violence

### Analysis

The current analysis builds on findings reported by Best and colleagues [7] showing that whilst men reported significantly more recovery strengths during their time in active addiction, this situation is later reversed with women reporting significantly more strengths in recovery, and thus greater growth of recovery assets in the period between active addiction and recovery. The aim of this paper is to investigate the specific domains of the recovery strengths and barriers scale (SABRS) across.

which this recovery capital growth occurs. Therefore, the paper adopts a three-step analysis approach.

First, we established if previous findings on gender differences [7] extend to our REC-PATH and RUN combined sample using independent t-tests that compared males and females on the four domain scores, as well as their increase in strengths and reduction of barriers in the period between active addiction and recovery. Second, we performed Chi-Square tests for independence in order to identify any significant associations between each SABRS item and the participant’s gender to address research question 1.

Third, we performed multiple linear regression analyses using the “forced entry” method to explore predictors of recovery capital growth in males and females separately in order to address research question 2. “Recovery capital growth” was calculated as the difference between recovery strengths and addiction strengths. Variables were declared “statistically significant” if *p* < 0.05 (i.e., working at 5% significance level). The variables included in the regression analyses were those SABRS items identified as significant in the second step of the analyses; including seven barrier items (have untreated emotional or mental health problems, have your driver’s licence revoked, damage property, been arrested, been charged with a criminal offence, been to prison, experience family violence); and twelve strength items (have a GP, have regular dental checks, take care of your health, maintain a driving licence, maintain a bank account, paid bills on time, maintain stable housing, remain in steady employment, further education or training, participate in family life, plan for the future, volunteer).

## Results

### Sample characteristics

A total of 1313 participants (combined over two studies) completed the LiR survey, including 854 males (65%) 453 females (34.5%) and six individuals (0.5%) who identified as ‘other’ gender (see Table 2). The mean age of the sample was 40.3 years (± 10.49), with a range of 18–74 years. The REC-PATH sample included individuals from the Netherlands (*n* = 231, 17.6%), Belgium (*n* = 181, 13.8%), and the United Kingdom (*n* = 364, 27.8%). The RUN international sample included participants from Serbia (*n* = 123, 9.4%), Poland (*n* = 79, 6%), Bosnia and Herzegovina (*n* = 72, 5.5%), Spain (*n* = 60, 4.6%), Croatia (*n* = 53, 4%), Sweden (*n* = 44, 3.4%), Montenegro (*n* = 15, 1.1%), Portugal (*n* = 6, 0.5%) and 85 (6.5%) individuals from other European countries. With regards to relationship status, the majority of participants were single and never married (*n* = 537, 40.9%), followed by married (*n* = 300, 22.8%), co-habiting (*n* = 213, 16.2%), divorced or separated (*n* = 198 = 15%), in other relationship situations (*n* = 48, 3.7%) and widowed (*n* = 17, 1.3%).

**Table 2:** Gender differences in recovery barriers and strengths

Mean number of	Male (***n*** = 854)	Female (***n*** = 453)	T, df, significance
Strengths in active addiction	4.7 (SD = 2.9)	4.5 (SD = 2.9)	1.3, 1305, *p* = 0.17
Barriers in active addiction	8.8 (SD = 3.3)	8.0 (SD = 3.1)	4.2, 1305, *p* < 0.001
Strengths in recovery	10.2 (SD = 3.4)	11.1 (SD = 2.7)	5.4, 1119.6, *p* < 0.001
Barriers in recovery	2.6 (SD = 2.4)	2.4 (SD = 1.9)	1.7, 1124.3, *p* = 0.08
Strengths change	5.4 (SD = 4.1)	6.5 (SD = 3.8)	5.0, 992.3, *p* < 0.001
Barriers change	−6.2 (SD = 3.9)	−5.6 (SD = 3.5)	2.7, 1013.8, *p* < 0.05

### Total recovery strengths and barriers by gender

Overall, participants reported a mean “increase” of 5.8 strengths (± 4.1) and a mean “reduction” of 6.0 (± 3.8) barriers between their periods in active addiction and recovery. Table 2 shows some notable gender differences; females (M = 11.1) showed significantly more strengths in recovery compared to males (M = 10.2) t = 5.4 *p* < 0.001; males (M = 8.8) have significantly more barriers in addiction compared to females (M = 8.0) t = 4.2 *p* < 0.001. The first key finding is that there was a significant difference on growth of recovery strengths in relation to gender; with females reporting a mean “increase” of 6.5 strengths (± 3.8) and males reporting a mean “increase” of 5.4 strengths (± 4.1) t = 5.0, 992.3, *p* < 0.001. No significant difference in the number of strengths in active addiction between males and females was identified.

### Associations between SABRS indicators at time of recovery and gender

The next step of the analysis identified any significant associations between each SABRS item at time of recovery and gender. Nineteen out of 32 items were significantly associated with gender; twelve out of those 19 significant associations were related to strengths at time of recovery, whilst the remaining seven significant associations were related to barriers at time of recovery, with the results shown in Table 3.

**Table 3:** Associations between SABRS items at time of recovery and gender

SABRS item			Males	Females	χ2 (df), significance
Strengths	General health management	Have a GP	87.2%	94.2%	25.39 [2], *p* < 0.001
		Have regular dental checks	61.0%	74.1%	23.23 [2], *p* < 0.001
		Take care of your health	82.2%	89.5%	12.12 [2], *p* < 0.01
	Daily life administration	Maintain a driving licence	77.5%	85.6%	10.19 [2], *p* < 0.01
		Maintain a bank account	86.2%	92.4%	11.74 [2], *p* < 0.01
		Able to pay bills	83.6%	90.7%	11.73 [2], *p* < 0.01
		Maintain stable housing	90.7%	92.6%	13.88 [2], *p* = 0.001
	Prosocial meaningful activities	Remain in stable employment	65.6%	76.5%	14.14 [2], *p* = 0.001
		Further your education or training	64.5%	76.9%	19.22 [2], *p* < 0.001
		Participate in family life	83.9%	92.2%	17.35 [2], *p* < 0.001
		Plan for the future	83.0%	89.3%	9.26 [2], *p* = 0.01
		Volunteer	61.9%	71.9%	12.54 [2], *p* < 0.01
Barriers	Mental health and wellbeing	Have untreated emotional or mental health problems	46.8%	51.6%	11.27 [2], *p* < 0.05
		Experience family violence	6.1%	10.4%	7.11 [2], *p* < 0.05
	Criminal Justice contact	Have your driver’s licence revoked	9.8%	2.0%	22.80 [2], *p* < 0.001
		Damage property	9.1%	4.3%	9.68 [2], *p* < 0.01
		Been arrested	10.2%	1.9%	34.81 [2], *p* < 0.001
		Been charged with a criminal offence	9.7%	1.2%	40.92 [2] *p* < 0.001
		Been to prison	7.0%	2.2%	14.57 [2], *p* = 0.001

Some of the highly significant gender differences in recovery (*p* < 0.001) include that significantly more women had a GP (94.2% vs. 87.2%) and regular dental checks (74.1% vs. 61.0%); maintained stable housing (92.6% vs. 90.7%); remained in steady employment (76.5% vs. 65.6%); furthered their education (76.9% vs. 64.5%); and participated in family life (92.2% vs. 83.9%) compared to men. However, significantly more women reported having untreated emotional or mental health problems (51.6% vs. 46.8%), and experienced family violence (10.4% vs. 6.1%) compared to men. We also found highly significant associations (*p* < 0.001) showing more males had their driving licence revoked (9.8% vs. 2.0%); were arrested (10.2% vs. 1.9%); were charged with a criminal offence (9.7% vs. 1.2%); and had been to prison (7.0% vs. 2.2%) compared to women. We have summarised these differences into broad life categories in Table 3.

### Factors associated with growth in strengths

For the multiple linear regression analyses, variables that were positively associated with increased “growth” in recovery strengths in all participants were: having regular dental checks; taking care of your health; paying bills on time; remaining in steady employment; furthering your education or training and volunteering. The only one variable negatively associated with “growth” (i.e., lower growth rates of recovery from addiction to recovery) was having your driver’s licence revoked. The analysis was repeated separately for males and females.

The increased “growth” model for males was highly significant (F (19, 383) = 27.76, *p* < 0.001), predicting 57% of the variance of growth in recovery strengths in men (see Table 4). The largest effects were found for prosocial meaningful activities (‘remain in steady employment’, ‘further your education’, and ‘volunteer’). The increased “growth” model for females was also significant (F (12, 180) = 6.13, *p* < 0.001) predicting 39% of the variance of growth in recovery strengths in women (see Table 5). The largest effects were found for general health management (‘take care of your health’) and one prosocial meaningful activity (‘furthered your education or training’). Due to some of the variables included having several missing cases, the totals for each gender differ in size and are considerably smaller than the overall sample.

**Table 4:** Multiple linear regression model of growth of recovery strengths in males

Predictor variables	B	standard error	Significance
Have a GP	−0.10	0.53	*P* = 0.85
Have regular dental checks	0.97	0.37	*P* < 0.05
Take care of your health	1.31	0.53	*P* < 0.05
Maintain a driving licence	−0.48	0.41	*P* = 0.25
Maintain a bank account	0.23	0.58	*P* = 0.68
Able to pay bills	1.37	0.63	*P* = 0.03
Maintain stable housing	0.47	0.63	*P* = 0.45
Remain in stable employment	1.62	0.38	*P* < 0.001
Further your education or training	1.83	0.39	*P* < 0.001
Participate in family life	1.18	0.58	*P* < 0.05
Plan for the future	−0.62	0.60	*P* = 0.30
Volunteer	2.72	0.37	*P* < 0.001
Have untreated emotional or mental health problems	−0.14	0.32	*P* = 0.66
Have your driver’s licence revoked	−1.42	0.55	*P* < 0.05
Damage property	0.86	0.65	*P* = 0.18
Been arrested	−0.06	0.96	*P* = .95
Been charged with a criminal offence	−0.41	0.91	*P* = 0.64
Been to prison	−0.31	0.71	*P* = 0.65
Experience family violence	0.04	0.70	*P* = 0.95

**Table 5:** Multiple linear regression model of growth of recovery strengths in females

Predictor variables	B	standard error	Significance
Constant	−1.31	1.45	*P* = 0.36
Have a GP	−1.05	0.89	*P* = 0.23
Have regular dental checks	0.50	0.60	*P* = 0.40
Take care of your health	2.88	0.91	*P* = 0.002
Maintain a driving licence	−0.71	0.77	*P* = 0.35
Maintain a bank account	1.08	1.01	*P* = 0.28
Able to pay bills	2.35	1.17	*P* < 0.05
Maintain stable housing	1.32	1.29	*P* = 0.30
Remain in stable employment	0.94	0.67	*P* = 0.16
Further your education or training	2.12	0.66	*P* = 0.002
Participate in family life	0.45	1.23	*P* = 0.71
Plan for the future	−0.35	0.99	*P* = 0.72
Volunteer	0.81	0.53	*P* = 0.13
Have untreated emotional or mental health problems	−0.00	0.45	*P* = 0.98
Have your driver’s licence revoked	−0.84	1.56	*P* = 0.59
Damage property	−1.79	1.41	*P* = 0.20
Been arrested	1.70	2.66	*P* = 0.52
Been charged with a criminal offence	3.24	4.08	*P* = 0.42
Been to prison	1.86	2.60	*P* = 0.47
Experience family violence	0.49	0.91	*P* = 0.59

## Discussion

The aim of the paper was two-fold; first, to identify any significant associations between SABRS items at time of recovery and gender and, second, to better understand the specific domains of recovery capital that best predict overall gains in recovery strengths according to gender in a large and geographically diverse European recovery sample. Findings showed that significantly more women than men in recovery report better general health management, daily life administration, and prosocial meaningful activities compared to men. However, women reported significantly higher rates around two ongoing barriers related to psychological health and domestic violence compared to men. In contrast, significantly higher rates of justice related barriers were found amongst men compared to women.

Subsequent analyses found different items were positively associated with increased “growth” in recovery strengths according to gender. Whilst one item relating to prosocial meaningful activities (‘further your education’) was found to significantly predict greater growth in recovery strengths for both men and women, two factors relating to meaningful activities (steady employment and volunteering) were most significant at predicting greater strengths growth amongst males. Finally, one item relating to general health management (‘take care of your health’) was most significant at predicting greater strengths growth amongst females. This is likely to have important implications for the supports provided by community recovery organisations and suggests that they should be gender-specific.

It has been suggested women have markedly different addiction and recovery careers compared to men [10, 16, 20, 22]. This study replicates previous research showing both that females report significantly more strengths in recovery compared to males (with women having on average 11 of the 15 strengths or resources measured in the Life in Recovery scale) based on a larger and more diverse sample than the previous finding, as well as a significant difference on growth of recovery strengths in relation to gender; with females reporting a greater mean “increase” of strengths compared to males [7]. Further, our findings extend previous research in three main ways.

First, we have identified that the items that best predict gains in recovery strengths in women emphasised general health management, consistent with Wincup (32), and furthering education or training. Furthermore, findings showed that although women in recovery report higher levels of general health management such as focusing on their health, daily life administration (such as paying bills and taxes) and better engagement with work and the community, they are more likely to have residual mental health problems and domestic violence experiences than men. This is both consistent with existing literature showing lower levels of wellbeing [27] and experiences of family violence amongst female participants [1, 24], but also novel given previous findings around financial difficulties among women in recovery [18] and lower levels of human capital compared to men [19]. It is important to highlight that although general health management was found highly significant in predicting female strengths “growth”, the rates of barriers relating to wellbeing (mental health and experiencing domestic violence) were higher among women compared to men. This supports the notion of overlapping barriers and facilitators as part of women’s experience of recovery [26]. Further, this finding supports previous research by Collinson and Hall [11] suggesting that some women report high levels of general health management yet they may continue to require ongoing support around mental health and gender-responsive services that are trauma informed. Our findings emphasise the challenge of positive recovery pathways in spite of managing residual problems relating to psychological health and trauma stemming from domestic violence for women in recovery [7].

Second, items that best predict gains in recovery strengths in men emphasised meaningful activities (remain in employment, furthering education and volunteering) supporting previous research in the UK [4] and the US [9] showing the importance of meaningful activities to building recovery capital. It is perhaps surprising, given previous suggestions about increased stigmatisation among women [21, 28], that women in recovery reported higher levels of full-time employment, furthering their education or training and volunteering during their recovery compared to men. This suggests that for some men, their overall recovery strength and wellbeing is impaired by failure to access sufficient meaningful activity, possibly due to the significantly higher rates of criminal justice related barriers reported by men compared to women in this sample. Research suggests individuals with previous involvement with the criminal justice system may experience economic disadvantage given their criminal records often prohibit gainful employment [14]. Finally, previous findings may be limited geographically, given the present study is one of the firsts to use a pan-European sample. However, what is clear is that, in this large European sample, barriers to recovery for men are more focused on justice and for women, on residual mental health and family violence factors. It is possible that observed differences between men and women are related to women leading more ‘law-abiding’ lives once in recovery, or that they have less residual criminal justice involvement from during their active addiction careers. Further, higher rates of untreated emotional or mental health problems amongst women may result in greater improvements as they continue on their recovery journeys given their greater use of services may provide a space for (re-)building themselves.

Third, two items relating to general health management (having regular dental checks; taking care of your health), one to daily life administration (paying bills on time) and three to prosocial meaningful activities (remaining in steady employment, furthering your education or training and volunteering) were found significant in predicting increased “growth” in recovery strengths for both men and women. Taking care of their health is important in predicting overall recovery strengths suggesting the importance of primary health engagement in addressing ongoing challenges with both ageing and the residual effects of substance using careers. The findings are both consistent with the existing literature on recovery careers and recovery capital in emphasising the importance of meaningful activities [4, 9] and positive health experiences, but also add something new in suggesting that women are typically more successful in engaging in these activities resulting in a greater growth of strengths in recovery. We also found evidence around the importance of professional development as the analyses indicated the item furthering your education or training is highly significant at predicting increased “growth” for both men and women, further contributing to the evidence around the added value of meaningful activities. In addition, it appears that it is detrimental to recovery to have difficulties related to transport as findings showed having your driver’s licence revoked was the only item that associated negatively with “growth” for both men and women. This supports previous research from the US suggesting mobility for employment as a relevant factor for recovery [9]. Indeed, the importance of access to transportation for those in their recovery journeys has been universally identified across addiction recovery research. Further, we note aspects relating to transportation being better addressed as one of many strengths of the SABRS.

There are significant limitations around sampling and representativeness. First, the study predominantly adopted a binary gender approach, and thus did not explore gender constructs other than men vs women. As a result, the study was not able to explore the recovery journeys of populations who identify themselves with a wider range of gender options, such as transgender individuals. Still, the study makes an important contribution to the discussion around gender specificity in recovery and to understanding pathways and predictors to strong recovery capital. We note the tendency to conflate gender and sex and emphasise the importance of enhancing our understanding around the complexity of the relationship between gender and recovery journeys. Second, we had no control over the sampling as it is entirely self-selected and thus includes no assessment of either the recovery status or previous experiences of the participants. Third, the study does not use a measure of culture to assess “cultural capital” as the SABRS aims to assess differences of cultural factors by identifying between-country differences. Fourth, there are some limitations common to the Life in Recovery model (see [3]), however the LiR has been established as an international instrument for monitoring recovery pathways. In addition, use of the LiR allows for access to participants outside of treatment populations and provides the opportunity to explore recovery pathways of individuals across a range of European settings. Our research uses the innovative method presented by Best and colleagues [7] which edits the LiR down to create an index of recovery capital called the Strengths and Barriers Recovery Scale (SABRS). However, it is important to acknowledge the SABRS scale remains relatively untested. Next, we recognise that the level of healthcare access available differs considerably in countries across Europe and beyond. Thus, we can make no conclusions about generalisability of our findings, but emphasise the importance of access to healthcare in the recovery journeys of both men and women. Finally, given that the sample in our study is exceptionally large, we are at risk of overpowering our findings. Thus, it is important to treat these findings with caution as these need to be replicated in future studies to establish their validity.

## Conclusion

The paper presents evidence on the significant differences across specific domains of recovery capital that best predict overall gains in recovery strengths according to gender. Findings showed items relating to prosocial meaningful activities were most significant when predicting strengths growth in men. In contrast, significantly higher rates of justice related barriers were reported by the males in this sample compared to women. In addition, whilst factors relating to general health management were found to be most significant when predicting greater strengths growth amongst women, our sample reported significantly higher rates in barriers related to psychological health and domestic violence compared to men. This study builds on previous research [7] by implementing the quantification of the Life in Recovery survey using a REC-PATH and RUN combined sample, as well as showing increased insight relating to differences in recovery pathways for males and females. Future research may explore possible explanations for the quantitative differences found in this study through qualitative research and novel research approaches (e.g., photovoice) [30]. Although more research is required to replicate the findings presented in this paper, our study points to the importance of the adoption of gender-specific approaches in recovery community organisations and the further study of differences in addiction and recovery careers according to gender to inform these.

## Data Availability

The raw data supporting the conclusions of this article will be made available by the authors, without undue reservation.

## Abbreviations

ARC: Assessment of Recovery Capital
LiR: Life in Recovery
REC-PATH: Recovery Pathways
RUN: Recovery Users Network
SABRS: Strengths and Barriers Recovery Scale
